# Improving consumer understanding of pesticide toxicity labels: experimental evidence

**DOI:** 10.1038/s41598-024-68288-9

**Published:** 2024-07-27

**Authors:** Hanin Hosni, Michelle Segovia, Shuoli Zhao, Marco A. Palma, Theodoros Skevas

**Affiliations:** 1 University of Delaware, Newark, USA; 2 University of Kentucky, Lexington, USA; 3 Texas A&M University, College Station, USA; 4 University of Missouri, Columbia, USA

**Keywords:** Environmental economics, Environmental impact, Psychology and behaviour, Socioeconomic scenarios

## Abstract

Consumers often inadvertently misperceive the health hazards associated with over-the-counter pesticides under the current textual labeling policy, potentially leading to improper use. We conducted an incentivized framed field experiment with eye tracking to evaluate the effectiveness of the current pesticide labels that convey risk using signal words (Caution, Warning, Danger) compared to two visually focused label alternatives: traffic light colors and skull intensity symbols. A total of 166 participants were randomly assigned to one of three label formats and asked to rank toxicity levels and make purchasing decisions within multiple price lists. Results show that signal words fail to adequately communicate toxicity levels. Specifically, participants’ correct assessment of toxicity level dramatically improves from 54% under the existing signal word label to 95% under the traffic light and 83% under the skull intensity symbol labels. We also find that participants are more likely to choose the less toxic alternatives under the new labels, suggesting the current labeling system may affect choice and have unintended adverse effects on human health.

## Introduction

In the United States, about 85% of households store at least one pesticide, with no required licensing to purchase general-use pesticides that can contain potentially hazardous substances^1^. The sheer volume of pesticide use, estimated at 1.1 billion pounds annually and accounting for an astounding 20% of the total global expenditure, emphasizes their ubiquitous presence and critical consequences of potential misuse^2^. Despite their prevalence and associated risks, consumers often lack the necessary information to understand the toxicity level of commonly used pesticides, potentially leading to improper handling and adverse health effects.

The pervasive use of pesticides is a double-edged sword. While they are developed to target weeds, insects, fungi, and rodents, the potential consequences to human health, pets, and the environment cannot be ignored^3^. The toxicity of certain chemical ingredients in pesticides, which are readily available to the public (without the requirement of any special training), has become a cause for concern. A well-known example is Glyphosate, the primary chemical ingredient in Bayer’s Roundup^®^, classified as a potential human carcinogen by the International Agency for Research on Cancer^4,5^. Similarly, other chemical substances found in pesticides, such as organophosphates, have been identified as posing significant threats to human health^6,7^. Previous studies suggested an association between pesticide exposure in households and different types of cancers and chronic diseases among adults^8–11^ and children^12,13^.

Although the United States implemented bans on chemicals such as PCBs (polychlorinated biphenyls) and DDT (dichloro-diphenyl-trichloroethane) many years ago^14,15^, pesticides containing toxic chemical ingredients still exist in the market, despite the proven linkage to health risks and being prohibited in other countries^16–18^. This raises critical questions about the role of pesticide labels in communicating risks to consumers and highlights the importance of labeling formats that effectively communicate the potential hazards to consumers so they can make more informed decisions. In this study, we evaluate consumer understanding of the existing signal words being used in pesticide labels to inform on toxicity level, comparing them to two alternative label prototypes, which are not yet available in markets. Our aim is to test for the effectiveness of these label formats in correctly communicating the toxicity level of pesticides, thereby contributing to more informed consumer pesticide choices.

Consumers’ understanding of the chemical contents in pesticides is limited^19–21^ and empirical studies related to commercial pesticide attributes and labeling are scarce. Evidence from previous research suggest that consumer awareness and understanding of the risks associated with pesticides influence their behavior. For instance, Bazoche et al. carried out experimental auctions to assess consumer willingness-to-pay for reducing pesticide use in apples and found a significant price premium for pesticide use reduction^22^. Rihn and Khachatryan surveyed 921 U.S. consumers on their awareness of pollinator-friendly plants and found that consumers with knowledge of neonicotinoid insecticides are more likely to purchase “neonic-free” plants^23^. Khachatryan et al. conducted an experiment with eye tracking to examine the effect of eco-labels on pesticide-free plants and found a positive correlation between pollinator-friendly attributes and consumers’ willingness to purchase. They also found that visual attendance positively influences consumers’ likelihood to purchase pollinator-friendly plants^24^.

The current Environmental Protection Agency (EPA) requirement for pesticide labeling operates under a seemingly straightforward but arguably ambiguous system. Labels utilize signal words such as “Caution,” “Warning,” and “Danger” to designate low, medium, and high levels of toxicity and potential hazards, respectively^25^. “Caution” indicates low toxicity; however, these products can cause slight eye or skin irritation and may be harmful if swallowed. “Warning” indicates a moderate level of toxicity, with the potential to cause moderate eye or skin irritation and harm if swallowed, inhaled, or absorbed through the skin. Substances labeled as “Danger” are highly toxic and carry severe risks, including the possibility of causing severe irreversible eye or skin damage. If the product is labeled “Danger” on the basis of its oral, inhalation or dermal toxicity (as distinct from skin and eye irritation), in addition to the label “Danger”, the word “Poison” must appear in red on a background of distinctly contrasting color, and the skull and crossbones symbol must appear in immediate proximity to the word “Poison”^25^. It is important to note that “Poison” is not a standalone (i.e., mutually exclusive) toxicity level under the US regulatory system (40 CFR 156.64). Hence in this study, we choose to focus only on the main three toxicity levels (Caution, Warning, and Danger). The semantic difference between these terms is subtle (particularly caution and warning), making it difficult for the public to discern the appropriate toxicity risk information^26,27^. This matter is of interest to public policy because general-use pesticides are available to the public without any required license, training, or education. The absence of regulatory oversight operates on the assumption that consumers read, comprehend, and adhere to the instructions and warnings contained within these commercially available pesticides and their safe disposal. However, for a pesticide label to be an effective risk communication tool, it must be clear and easily understood by consumers^28^. The terms ‘pesticides’ and ‘herbicides’ are used interchangeably in the paper because labeling regulations in the U.S. are the same for all pesticide products (herbicides, insecticides, fungicides, etc.)^25^.

We conducted a framed field experiment with a sample of 180 consumers who were randomly presented with one of three formats of human hazard labeling: (1) a signal word display, which aligns with the status quo serving as the baseline; (2) a traffic light display, employing universally recognized color cues; and (3) a visual skull intensity symbol display, designed to capture attention and convey severity. We examine to what extent consumers correctly assess the toxicity levels under each labeling format, and how this influences their pesticide choices. Furthermore, we elicit consumers’ willingness-to-pay for lower toxicity product alternatives relative to the more dangerous counterpart under each labeling format.

Research in cognitive psychology suggests that visual information, such as images and graphics, is superior in conveying information effectively compared to words^29,30^. This principle is especially relevant to pesticide labels, where complex toxicity information needs to be communicated quickly and clearly. Moreover, findings from studies in marketing and psychology suggest that consumers are particularly responsive to visual elements, such as colors and symbols, when it comes to capturing their attention^31–33^. For example, graphic logos such as traffic lights and three-tiered stars are associated with improved purchase behavior that emphasizes environmental and health concerns^34–36^. Hence, the prototype labels are designed to test the effectiveness of graphical and visual elements at conveying toxicity information.

Our findings suggest that labels using a traffic light and a skull intensity symbol drastically improve the ability of respondents to accurately assess toxicity levels. The magnitude of the effect is big. While only 54% of participants accurately distinguish the toxicity level under the existing signal word display (basline condition), this percentage increases to 95% under the traffic light display and 83% under the skull intensity symbol display, which in turn steer them towards choosing a less toxic pesticide alternative. Further, participants exhibit significantly lower price premiums for the less toxic pesticide under the current signal word labels ($1.8) compared to the suggested alternative labels ($4.8 and $3.3 under traffic light and skull intensity symbol labels, respectively). These findings suggest that due to the lack of clarity of the signal word labels to convey toxicity levels, participants are less likely to choose the less toxic pesticide and exhibit price discounts for these products.

Our findings provide policymakers with actionable evidence to improve labeling regulations and reduce health risks. This, in turn, may lead consumers to make more informed choices and potentially choose more health-friendly pesticides. Additionally, our findings offer guidance to manufacturers, retailers, and consumer advocacy groups seeking to promote informed decision-making and product safety within the pesticide market.

The rest of the paper is organized as follows. The next section describes the experimental design and procedures used to collect data. Section three discusses the results, and section four concludes and provides policy implications.

## Methods

The experiment consisted of a between-subject design, in which participants were randomly assigned to one of three treatments with the label display format as a key treatment factor: (i) signal word display or baseline, (ii) traffic light display, and (iii) skull intensity symbol display. A summary of the experimental design is presented in Table 1. Label formats were presented as follows:

**Table 1:**
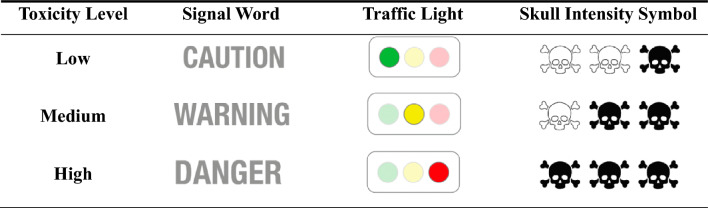
Health hazard labeling design under different experimental treatments

*Signal word display (status quo or baseline)* Subjects were exposed to conventional pesticide labels presenting the health hazards through signal words including “Caution,” “Warning,” and “Danger.” In compliance with the U.S. EPA pesticide labeling standards, these terms correspond to low, medium, and high toxicity levels, respectively.

*Traffic light display* This format utilized a color-coded system (green, yellow, and red) to denote low, medium, and high toxicity levels, respectively.

*Skull intensity symbol display* Each toxicity degree was symbolized by one, two, or three skull-crossbones contours filled in, reflecting the toxicity intensity. For instance, a low toxicity level would be denoted by coloring one skull, a medium level by coloring two skulls, and a high level by coloring all three skulls.

### Sample

The sample consists of 180 subjects from the general population of the Southwestern region of the United States. The sample size was determined based on a power analysis with 0.8 power, a medium effect size, and a 5% significance level. Participants were recruited through bulk emails sent to an existing university’s participant database and advertisements posted in local newspapers. Subjects received $20 as compensation for their participation. The experimental sessions were conducted in spring 2020 and spring 2021 at different times of the day (data collection was suspended in fall 2020 due to the Covid-19 pandemic and resumed spring of 2021). Each session had between 10 and 15 participants and lasted approximately 30 min. The study was approved by the Institutional Review Board and all methods were performed in accordance with the relevant guidelines and regulations.

### Experimental procedures and design

Upon arriving at the lab, participants signed a written informed consent form. They were then seated in front of the computer station, and their eye movements were calibrated prior to initiating the experimental tasks. All tasks were computerized. Subjects completed several tasks; however, this paper includes only the results from the multiple price lists (MPLs) and the post-experimental questionnaire. All subjects completed the MPL tasks first. Participants were informed that the computer would randomly select one of the tasks at the end of the session to be binding, and that there is a 10% chance to implement their decisions with real payments for the binding task. In this regard, Ahles et al. (2024) found that incentivized schemes with 10% payment probability are effective in eliciting valuations that are statistically indistinguishable from a fully incentivized scheme^37^.

To elicit whether participants can properly assess the risk level displayed in the pesticide labels, in each treatment, we asked them to rank the toxicity level of the corresponding label format from the least to the most toxic. We then examine if participants’ assessment of the toxicity level displayed in labels impacts their pesticide choice and willingness to pay for pesticides with varying toxicity levels (images of pesticides alternatives were identical in everything except their toxicity level. No information about product quality/effectiveness was given to participants). To do so, participants completed three multiple price lists (MPL) tasks, each comparing two pesticides differing in their toxicity levels: high toxicity vs. low toxicity, high toxicity vs. medium toxicity, and medium toxicity vs. low toxicity. Each MPL consisted of 12 binary choices between two pesticides with varying toxicity levels and prices, allowing us to estimate the tradeoffs consumers make between toxicity levels and cost. Hence, each participant completed a total of 36 choice sets. Each choice set was presented separately on the computer screen. In each choice set, the price of the more toxic pesticide was fixed at $10, while the price of the less toxic pesticide started at $20 in choice set 1 and decreased by $1 in the following choice sets. That is, in choice set 10, the more toxic pesticide was $1 cheaper than the less toxic option, in choice set 11 the price was identical for both pesticide alternatives, and in choice set 12 the less toxic pesticide was $1 cheaper than the more toxic alternative. If both pesticides have similar effectiveness, subjects are expected to switch to the less toxic pesticide at choice set 11 (when both products are priced equally). In our analysis, we calculate subjects’ willingness-to-pay to switch from a more toxic pesticide to a less toxic pesticide product based on their switching point in the MPL task. Out of the 180 responses, 14 observations were excluded from the analysis due to multiple switching behavior. Multiple switching behavior is often used as indicative of indifference/confusion between the choices; therefore, it is common practice to remove this data from the analysis^38,39^. Also, none of our subjects exhibited a never switching behavior. Hence, our final sample comprises 166 participants. An example of the label formats used in each treatment, and a table summarizing the MPL task, is added in the supplementary materials (Tables 1 and 2, respectively).

As a real incentivized experiment, respondents were given a $10 endowment at the beginning of each MPL. At the end of the session, if the MPL was randomly selected as the binding task, subjects had a 10% chance to purchase the product chosen in the binding decision at the corresponding market price. If the market price was higher than the $10 endowment, then subjects covered the difference in price from the $20 participation fee. Finally, subjects filled out a questionnaire regarding their socio-demographic characteristics (i.e., age, gender, race, education level and, household income) and experience using pesticide products.

### Eye-tracking measures

Throughout the experiment, subjects’ eye movements were recorded using a Tobii TX300 eye tracking device (Tobii 2014). The eye tracker was embedded in the computer and tracked gaze position using near-infrared technology at a sampling rate of 120 Hz (i.e., 120 data points per second). The height of the desk with the computer was adjusted, and a nine-point eye calibration was performed to ensure gaze accuracy and precision. Moreover, the light conditions in the lab were kept constant across sessions.

**Figure 1:**
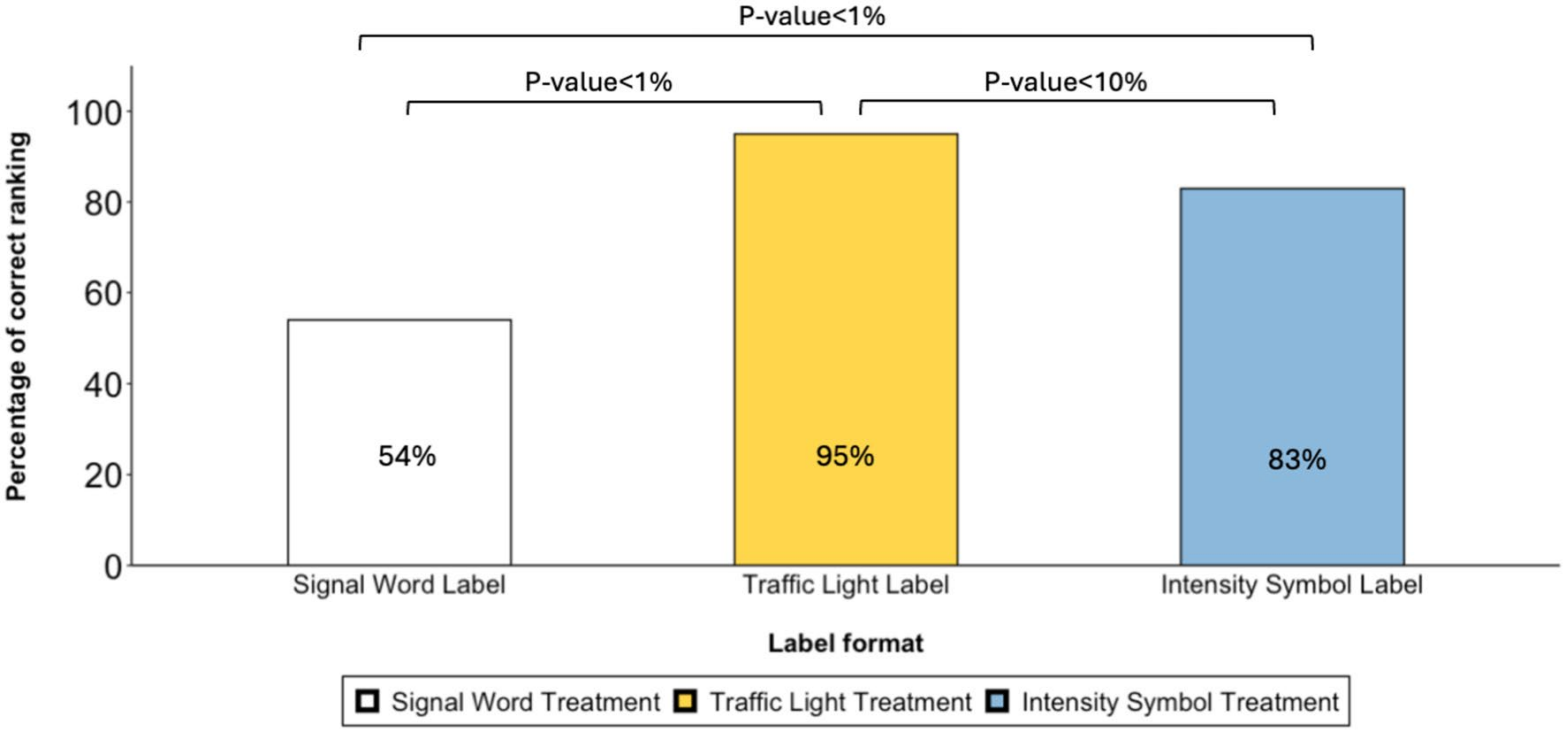
Percentage of participants who correctly ranked toxicity levels in each labeling format by treatment. *p*-value < 1% and *p*-value < 10% indicate significance at levels 1% and 10% respectively per Mann–Whitney U test.

We use total visit duration (TVD) as a measure of participants’ visual attention while performing the experimental tasks (while eye tracking data were collected throughout the experiment, we report eye tracking metrics only for the MPL task as it is the main outcome of this paper). To obtain this metric, we create two separate areas of interest (AOI), each covering the health hazard label area for each pesticide option. TVD is the amount of time in milliseconds (based on fixations) that the subject spends viewing an AOI (i.e., label area). This eye-tracking metric has been validated as a measure of respondents’ visual attention to a stimulus^40–46^. An example of an AOI is shown in the supplementary materials (Fig. 1).

## Results and discussion

Table 2 provides the descriptive statistics for several socio-demographic measures obtained in the questionnaire. Using Kruskal–Wallis tests, we find no statistical differences in the subject characteristics across treatments (*p* > 0.10 for all tests), suggesting that our randomization was effective. On average, 41% of the sample is male, with an average age of 29 years and household income of $74,458. About 45% of subjects identify themselves as White and 55% have a college degree.

**Table 2:** Summary statistics by treatment.

Characteristics	Overall, N = 166^a^	Signal word N = 57^a^	Traffic light, N = 56^a^	Skull intensity symbol, N = 53^a^	Kruskal–Wallis *p*-value
Age	29 (13)	31 (15)	27 (11)	30 (13)	0.3
Male	41%	47%	41%	34%	0.4
White	45%	39%	41%	57%	0.13
College	55%	58%	52%	57%	0.8
Income	74,458 (47,800)	74,912 (51,352)	73,571 (45,083)	74,906 (47,530)	0.9

^a^Mean (SD)/N (%).

To examine subjects’ assessment of the toxicity level, in each treatment, they were asked to rank the toxicity levels of the corresponding label format from the least to the most toxic. In the signal word treatment, only 54% of the participants correctly ranked the signal word labels. However, in the traffic light treatment, 95% accurately ranked the traffic light labels, while 83% of participants in the intensity symbol treatment correctly identified the intensity symbol format (Fig. 1). This result confirms that visual information, utilizing colors and images, is more effective at conveying toxicity levels than signal words alone. Pictorial labels are clearer and easier to understand compared to signal word labels. Easy and clear risk communication is essential for informed decision-making and promoting safe product use.

**Figure 2:**
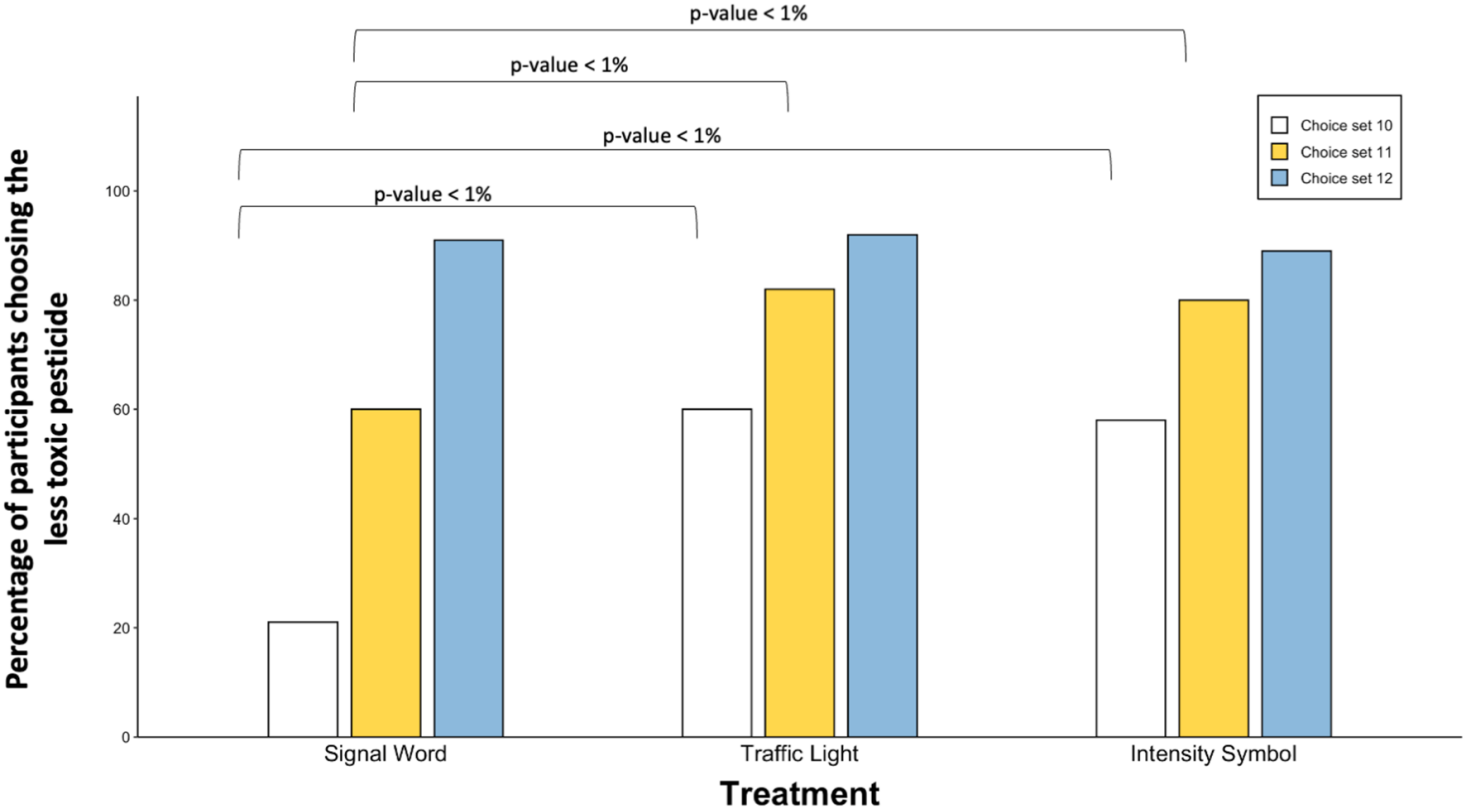
Percentage of participants selecting the less toxic pesticide under each choice set across treatment. Choice set 10: more toxic pesticide is $1 cheaper; Choice set 11: both pesticide options are equally priced; Choice set 12: less toxic pesticide is $1 cheaper. *p*-value < 1% indicates significance at 1% level per Mann–Whitney U test.

We next show that when faced with several pesticide alternatives, subjects’ lack of understanding of the current EPA signal word-based labels affects their pesticide choices. We begin by estimating the percentage of subjects selecting the less toxic pesticide in choice sets 10, 11, and 12 by treatment (these estimates are aggregate mean percentages in terms of toxicity level). Results from Fig. 2 show that in choice set 10, when the more toxic pesticide is $1 cheaper than the less toxic counterpart, only 21% of subjects select the less toxic option under the standard signal word format. Comparatively, this percentage is significantly higher in the traffic light (60%) and the skull intensity symbol (58%) formats. In choice set 11, where both pesticide options are priced equally, 60% of subjects choose the less toxic pesticide under the signal word format compared to around 80% under the other two display formats. On the other hand, in choice set 12, where the less toxic pesticide is $1 cheaper than the more toxic counterpart, most subjects (94%) opt for the safer pesticide. One could hypothesize that toxicity level could be an indicator of the effectiveness that people use when purchasing pesticides. While we acknowledge that some people may prefer a product with higher toxicity because they perceive it to be more effective, we do not examine the relationship between toxicity and product performance as our objective is to determine whether people can distinguish toxicity levels under the current EPA label format compared to the suggested label prototypes.

Table 3 reports price premiums for the less toxic option by toxicity level. When comparing pesticides with the largest difference in toxicity level (high vs low), we find that subjects are willing to pay a price premium of $1.3 for the less toxic pesticide under the signal word format. On the other hand, the premium increases by more than three times in the traffic light treatment to $4.8 and increases to $3.3 in the skull intensity symbol treatment. As the toxicity difference between the two options decreases, the premium decreases. However, across all toxicity levels, the price premiums for the less toxic product are significantly larger under the traffic light and the skull intensity symbol formats compared to signal word (*p* < 0.001 for all tests). We also find that the price premiums of subjects who correctly ranked the toxicity levels in the signal word format are greater than those who ranked them incorrectly; although this effect is evident across all toxicity levels, it was only significant (*p*-value < 0.1) when comparing pesticides with the largest difference in toxicity level (high vs low) (see Table 3 in the supplementary materials). The results reported in Table 3 are robust to linear panel regressions with and without controlling for observables (see Table 4 in the supplementary materials).

**Table 3:** Price premium for the less toxic option by toxicity level.

Toxicity level	Signal word, N = 57 Mean (SD)	Traffic light, N = 56 Mean (SD)	Intensity symbol, N = 53 Mean (SD)	Kruskal–Wallis *p*-value
High–Low	1.3 (2.7)	4.8 (4.2)	3.3 (3.7)	< 0.001
High-Medium	1.2 (2.9)	3.6 (3.9)	2.4 (3.1)	< 0.001
Medium–Low	0.8 (2.3)	2.7 (3.5)	2.2 (2.9)	< 0.001

**Table 4:** Time spent (milliseconds) on each labeling design.

Toxicity level	Overall, N = 2585^a^	Signal word, N = 845^a^	Light, N = 945^a^	Symbol, N = 795^a^	*p*-value^b^ (SW vs L)	*p*-value^b^ (SW vs S)	*p*-value^b^ (L vs S)	*p*-value^c^
High	962 (1538)	1075 (1561)	951 (1713)	853 (1262)	< 0.001	0.005	0.123	< 0.001

Toxicity level	Overall, N = 2800^a^	Signal word, N = 845^a^	Light, N = 1053^a^	Symbol, N = 902^a^	*p*-value^b^ (SW vs L)	*p*-value^b^ (SW vs S)	*p*-value^b^ (L vs S)	*p*-value^c^
Low	819 (1263)	995 (1345)	755 (1250)	728 (1180)	< 0.001	< 0.001	0.686	< 0.001

^a^Mean (SD).

^b^Mann–Whitney U test.

^c^Kruskal–Wallis rank sum test.

Finally, we test whether subjects’ visual attention towards the different label formats has an impact on their pesticide choices. We use total visit duration (TVD) as a metric for visual attention towards each label in the high toxic option and the low toxic counterpart by treatment. Results from Table 4 indicate that subjects spend significantly higher time on the signal word labels than on the traffic light and the skull intensity symbol labels (*p* < 0.001 for all tests). This result seems to point out that there is more effort required and hence a larger amount of time spent trying to understand the information presented in the technical words used to highlight the toxicity level of the product. This explanation aligns with previous work showing that more difficult decisions require a greater amount of time for deliberation and resolution^47^.Recall that only 54% on the sample ranked the toxicity levels correctly under the current labeling format compared to 95% in the traffic light and 83% in the skull intensity symbol formats.

Moreover, the increase in visual attention towards the more toxic option translates into a higher proportion of subjects choosing that alternative under a signal word format. This effect is not observed in the traffic light and skull intensity symbol treatments where most subjects choose the less toxic alternative despite paying more attention toward the counterpart. This provides some evidence that the labels are affecting the difficulty, deliberation, and choice process^48–51^. In sum, results from eye tracking data show that although participants spend more time looking at the signal word labels (to process a more difficult label), traffic light and skull intensity symbol formats are more effective in motivating the choice of less toxic pesticide options.

## Conclusions

Pesticide labels ensure the safety of usage and prevent potential health risks. However, our study reveals that the current U.S. EPA pesticide labeling system, with its reliance on signal words “caution,” “warning,” and “danger” to convey information about the low, medium, or high toxicity of the products, may not be able to effectively communicate toxicity risks to consumers. Per existing research, in terms of communicating toxicity risk information, such written descriptions are of questionable effectiveness^26,27^. In fact, Merriam-Webster lists “caution” and “warning” as synonymous^52^. This research responds to the urgent need to test different pesticide labeling policies to prioritize consumer understanding and safety.

We implemented an incentivized framed field experiment to test the effectiveness of two alternative label prototypes with visial elements on pesticide choices: a traffic light that uses color-coded system and a skull intensity symbol consisting of a pictorial design. Our findings suggest that the traffic light and skull intensity symbol labels drastically improve the ability of respondents to accurately assess the toxicity level, gravitating them towards a less toxic pesticide alternative. In fact, while only 54% of participants can accurately distinguish the toxicity level from the existing signal word-based label, this percentage increases to 95% in the traffic light and 83% in the skull intensity symbol labels.

Importantly, participants are more likely to choose the less toxic alternatives under the new labels. In terms of product valuation, we find that under the current signal word labels, participants exhibit significantly lower price premiums for the less toxic pesticide compared to the suggested label alternatives. Interestingly, our results also reveal that while participants spend a greater amount of time looking at the signal word labels, the alternative formats are more effective in influencing choices toward less toxic pesticides. Our findings emphasize the effectiveness of visual aid elements in the labeling formats such as traffic lights and intensity symbols, aligning with previous studies suggesting simplicity and clarity as means of improving the understanding of labeling information^53^. While our study considers only two alternative label formats, there may be other possible alternatives, such as combining text and pictorial labels or the use of simpler text labels. Also, the pesticide labels currently available in the market contain additional information that could further impact consumer understanding and decision-making. Future research could examine how label complexity, including ingredient lists, can influence pesticide choice and valuation.

Our finding suggests that a transition towards more informative and visually intuitive labels, such as traffic lights or image-based systems, could empower consumers to make more discerning decisions and minimize exposure to potentially hazardous chemicals in pesticides. Such a shift also enhances consumers’ ability to identify risky ingredients more readily. It is important to note that the traffic light system may be less effective for populations with specific conditions (e.g., color blindness) and this issue should be considered in labeling design. In implementing labeling changes, it is important for policymakers to work with pesticide manufacturers to foster compliance and facilitate the smooth adoption of effective label designs that align with both public health objectives and industry standards.

In conclusion, our study emphasizes the urgent need to rethink pesticide labeling. This renewed approach must prioritize not only consumer safety but also broader societal considerations, fostering an environment that is both healthy and informed. The evidence provided in this research underscores the importance of a comprehensive shift in labeling policies, moving beyond traditional textual information, and embracing innovative consumer-friendly alternatives.

### Supplementary Information

Supplementary Information.

## Data Availability

Data will be provided if requested. Please contact Dr Hanin Hosni at hosnih@udel.edu.
