# Supplementary material for: Improving consumer understanding of pesticide toxicity labels: experimental evidence
Source: Sci Rep. 2024 Jul 27;14:17291. doi: 10.1038/s41598-024-68288-9 (PMC11283515; doi:10.1038/s41598-024-68288-9)
Supplement: Supplementary file 1 — Supplementary Information. [file 41598_2024_68288_MOESM1_ESM.docx]

**Improving consumer understanding of pesticide toxicity labels:**

**experimental evidence**

***Corresponding author:***

Hanin Hosni [hosnih@udel.edu](mailto:hosnih@udel.edu) University of Delaware

***Co-authors:***

Michelle Segovia [segoviam@udel.edu](mailto:segoviam@udel.edu) University of Delaware

Shuoli Zhao [szhao@uky.edu](mailto:szhao@uky.edu) University of Kentucky

Marco A Palma [mapalma@tamu.edu](mailto:mapalma@tamu.edu) Texas A&M University

Theodoros Skevas [skevast@missouri.edu](mailto:skevast@missouri.edu) University of Missouri

**Supplementary Materials**

**Table 1.** Labeling format under each treatment for choice scenario High Toxicity-Low Toxicity


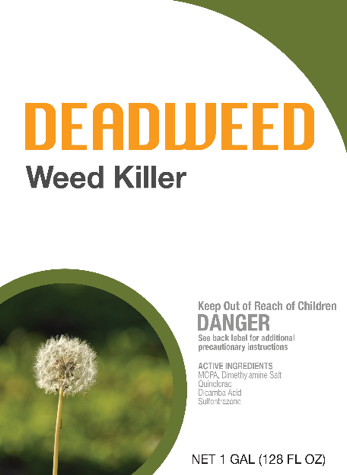

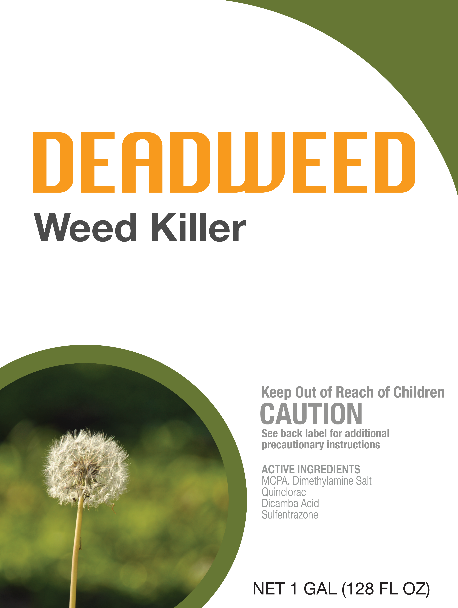


| **Signal Word** |  | |
| --- | --- | --- |
| **Traffic Light** |  | 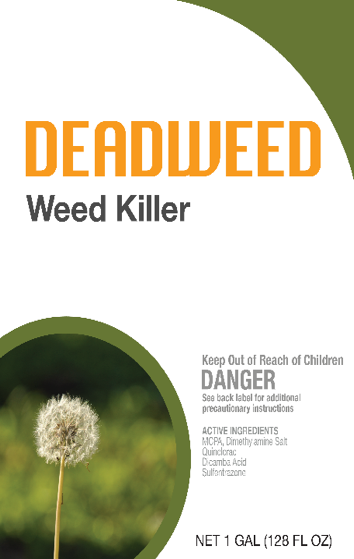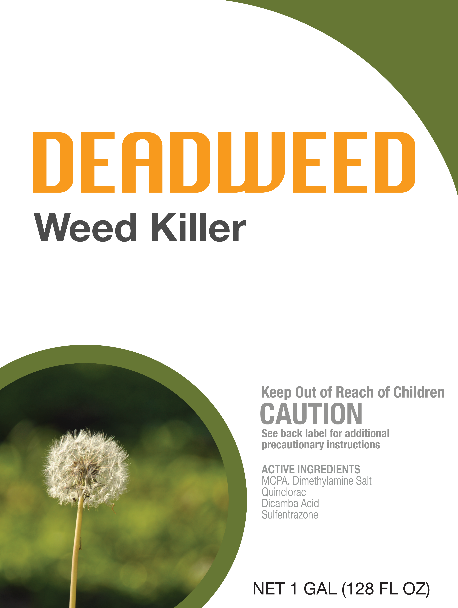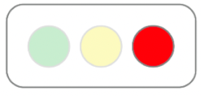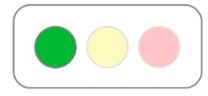 |
| **Skull Intensity Symbol** | 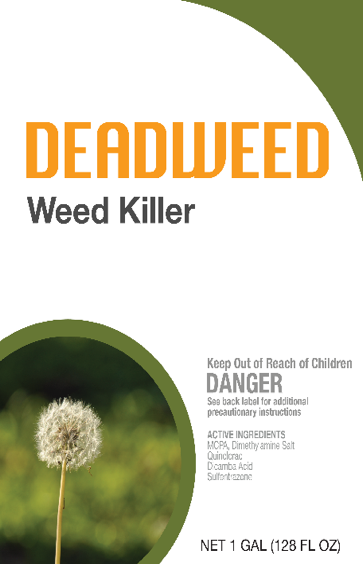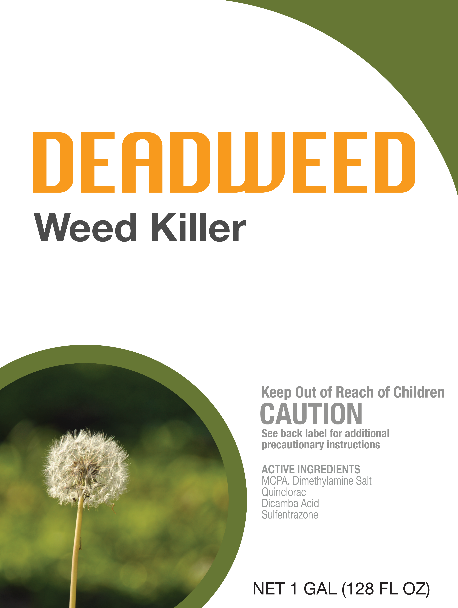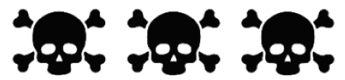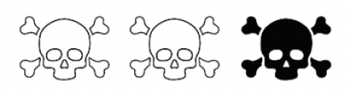 |  |

**Table 2.** Herbicide choice sets under the signal word treatment for choice scenario High Toxicity-Low Toxicity

| **Choice Set** | **Option A** | | **Option B** | |
| --- | --- | --- | --- | --- |
|  | **Price** | **Toxicity** | **Price** | **Toxicity** |
| 1 | $10 | DANGER | $20 | CAUTION |
| 2 | $10 | DANGER | $19 | CAUTION |
| 3 | $10 | DANGER | $18 | CAUTION |
| 4 | $10 | DANGER | $17 | CAUTION |
| 5 | $10 | DANGER | $16 | CAUTION |
| 6 | $10 | DANGER | $15 | CAUTION |
| 7 | $10 | DANGER | $14 | CAUTION |
| 8 | $10 | DANGER | $13 | CAUTION |
| 9 | $10 | DANGER | $12 | CAUTION |
| 10 | $10 | DANGER | $11 | CAUTION |
| 11 | $10 | DANGER | $10 | CAUTION |
| 12 | $10 | DANGER | $9 | CAUTION |


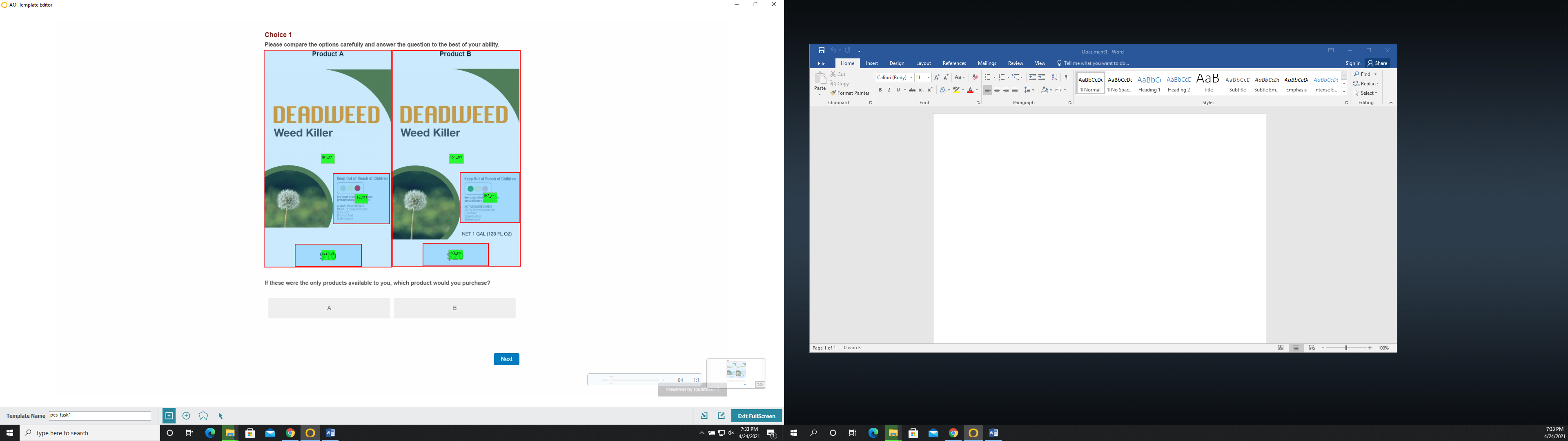


**Figure 1.** Example of AOI

**Table 3.** Effect of signal word labels understanding on the premium of the less toxic option by toxicity level

| **Toxicity level** | **Signal Word Right,** N=31  Mean (SD) | **Signal Word Wrong,** N=26  Mean (SD) | **Mann-Whitney U**  **p-value** |
| --- | --- | --- | --- |
| High-Low | 1.8 (3.0) | 0.76 (2.3) | 0.06 |
| High-Medium | 1.3 (3.0) | 1.2 (2.7) | 0.7 |
| Medium-Low | 0.9 (2.4) | 0.72 (2.2) | 0.6 |

Note: Signal Word Right refer to participants who ranked the signal word labels correctly; Signal Word Wrong refer to participants who ranked the signal word labels incorrectly.

**Table 4.** Impact of label design on the premium of the less toxic option

| **Independent Variable** | **(1)** | **(2)** | **(3)** |
| --- | --- | --- | --- |
| Signal word | -1.49 (0.53) ^***^ | -1.13 (0.57) ^*^ | -0.96 (0.57) |
| Light | 1.06 (0.63) ^*^ | 1.21 (0.67) ^*^ | 1.26 (0.58) ^**^ |
| High-Low | 0.74 (0.16) ^***^ | 0.96 (0.27) ^***^ | 0.96 (0.27) ^***^ |
| Medium-Low | -0.53 (0.17) ^***^ | -0.23 (0.21) ^***^ | -0.23 (0.21) ^***^ |
| High-Low*Signal word |  | -0.89 (0.35) ^**^ | -0.89 (0.35) ^**^ |
| High-Low*Light |  | 0.25 (0.40) | 0.25 (0.40) |
| Medium-Low* Signal word |  | -0.19 (0.34) | -0.19 (0.34) |
| Medium-Low*Light |  | -0.70 (0.43) ^*^ | -0.70 (0.43) ^*^ |
| Pesticide label familiarity |  |  | 0.26 (0.32) |
| Age |  |  | -0.04 (0.02) |
| Male |  |  | -0.98 (0.47) ^**^ |
| White |  |  | 0.24 (0.51) |
| Income |  |  | -0.05 (0.04) |
| College |  |  | 0.41 (0.55) |
| Constant | 2.55 (0.42) ^***^ | 2.38 (0.42) ^***^ | 3.27 (1.00) ^***^ |
| Observations | 498 | 498 | 498 |
| Adjusted R^2^ | 0.13 | 0.15 | 0.16 |
| F statistics | 75.49^***^ | 96.89^***^ | 106.27^***^ |

Notes: Reported values are the estimated coefficients. Single, double, and triple asterisks (*, **, ***)

indicate significance at the 10%, 5%, and 1% levels. Values in parenthesis are robust standard errors.
